# Research on blasting technology for thick and hard roof fracturing in isolated island coal mine working face

**DOI:** 10.1038/s41598-025-98911-2

**Published:** 2025-04-25

**Authors:** Wei Huang, Gang Jing, Liqiang Ma, Leilei Zhao, Shuai Wang

**Affiliations:** 1 Key Laboratory of Xinjiang Coal Resources Green Mining, Ministry of Education, Xinjiang Institute of Engineering, Urumqi, 830023 China; 2 Chen Jiashan Coal Mine, Shaanxi Shanmei Tongchuan Mining Co., Ltd, Tongchuan, 727200 China; 3 Xinjiang Key Laboratory of Coal-Bearing Resources Exploration and Exploitation, Xinjiang Institute of Engineering, Urumqi, 830023 China; 4 Xinjiang Engineering Research Center of Green Intelligent Coal Mining, Xinjiang Institute of Engineering, Urumqi, 830023 China

**Keywords:** Coal burst, Roof blasting, Drilling pattern, Infrared radiation, Effect test, Natural hazards, Solid Earth sciences

## Abstract

In order to study the prevention and control technology for hard roof type coal burst in the isolated working face of the Chenjiashan coal mine, the 418 isolated working face was selected as the engineering case study. Based on the different impact danger zones and mining areas, a roof breaking blasting pressure relief technical scheme was proposed. The anti-impact effect was verified through hole peeping and infrared radiation data. The research shows: (1) According to the geological conditions of the 418 working face of the Chenjiashan coal mine, the working face is divided into weak impact hazard areas and moderate impact hazard areas. Targeted roof blasting schemes were proposed for the initial square area of the working face, moderate danger area, weak danger area, initial mining and initial caving area, and the strike area of the working face. (2) On-site borehole data show that after blasting, a large number of fractures and delaminations were formed in the roof, and some fractures further developed into delaminations, with local areas showing crushed zones. This proves the formation of a “buffer zone” in the roof and floor, achieving pre-cracking of the thick and hard roof, full development of fractures, significant reduction in stress concentration, and the roof blasting can achieve good pressure relief effect. (3) The temperature monitoring near the blasting point and the infrared radiation temperature shows that within an hour after the implementation of the roof blasting, the coal mass at the breaking position experienced a process of heating up and then cooling down, with the temperature at the monitoring point rising by 0.5–0.7 °C. After the implementation of the roof blasting, the key layer above the working face was destroyed, and the stress was released and transmitted to the corresponding area of the coal mass, the stress of the coal increased, and the infrared radiation temperature increased, proving that the blasting pressure relief achieved the expected effect.

## Introduction

Coal burst is a dynamic phenomenon that occurs when the coal and rock masses surrounding a roadway or working face accumulate elastic energy due to mining disturbances, ultimately leading to a sudden release of elastic strain energy and causing severe damage. Essentially, it is the sudden instability and failure of coal and rock masses under high ground stress^1,2^. A thick and hard roof is the strata located above the coal seam that is characterized by high strength, large thickness, and resistance to collapse. The high-strength mining pressure it exerts has always been an important factor affecting the safety of coal mines and the efficient extraction of coal. It is also one of the main causes of coal bursts^3,4^.

Among them, the isolated island face is a special working face surrounded by mined-out areas, leading to more complex stress distribution and significantly higher stress concentration than ordinary faces. Its unique geometry also results in less predictable stress transfer and a higher likelihood of sudden energy release, increasing the risk of coal burst and posing greater challenges for prevention and control.

A number of scholars have conducted extensive research on the mechanisms of coal bursts^5,6^, as well as monitoring and early warning methods^7–9^. However, the existing mechanisms of coal bursts are unable to fully explain the phenomenon. Additionally, most current field monitoring methods primarily focus on ‘monitoring only, without early warning’, which limits their effectiveness in preventing and controlling coal bursts at the site. Therefore, taking measures to relieve roof stress during coal mining to prevent the occurrence of coal bursts has become the main method of anti-impact protection^10,11^.

Roof blasting technology, as a method of blasting within the roof rock layer without a free face for blasting, weakens the structural strength of coal and rock mass in specific areas, thereby reducing the risk of impact hazards^12^. For example, Yang et al.^13^ conducted research on the directional fracturing blasting pressure relief technology for dynamic pressure roadways, elaborated on the mechanical mechanism of directional blasting and the relevant calculation formulas, and monitored and analyzed the deformation of the roadway before and after pressure relief. Liu et al.^14^ based on the principle of stress relief caused by the construction of overlying rock strata and the cutting of the roof, established a mechanical model of the triangular arch structure and a discrete element model of the surrounding rock movement, revealing the stress relief effects of roof blasting and cutting and their impact on the stability of tunnel support. Tang et al.^15^ analyzed the parameters of blasting holes and, through underground blasting experiments, studied the impact of the charging structure and the amount of explosives on the blasting effect. Meng et al.^16^ proposed an optimized blasting design for the control of hard roof during the early mining stage of a mine, indicating that the new design using a serrated blasting hole overcame the disadvantages of traditional stress relief blasting. Kong et al.^11^ established expressions for the lateral support stress distribution near the end of the working face before and after roof cutting through theoretical analysis. They used numerical simulation methods to analyze the stress and plastic zones in the mining area, as well as the deformation characteristics of the tunnel after roof cutting.

In recent years, an increasing number of scholars have utilized infrared radiation to study the mechanical properties of coal and rock masses^17–19^. During the mining process at the working face, the coal-rock mass will produce a large number of fractures due to disturbance. If fractures and defects appear on the surface of the coal-rock mass, the infrared radiation from the surface will undergo significant changes^20–22^. Some scholars have studied the temperature change patterns of infrared radiation during the fracturing process of coal-rock bodies. For example, Cao et al.^23^ researched the patterns of infrared radiation changes during the cyclic loading and unloading process, and they believe that there is a characteristic of "abrupt change—rapid decline" in the average infrared radiation temperature of rocks during the later stages of loading and unloading. Wei et al.^24^ proposed using the damaged infrared energy response index to track the evolution of cracks and to identify precursors to fracturing. Yin et al.^25^ studied the different damage states and failure modes of coal under different types of impacts and obtained the infrared radiation and acoustic emission precursor characteristics of different types of coal failure effects. Li et al.^26^ used an infrared thermography camera to monitor the damage and failure process of coal-rock composites in real-time and established a numerical model for the destabilization and damage of coal-rock. Lin et al.^27^ compared the changes in damage morphology and mechanical property indices before and after high-temperature treatment, and analyzed the evolution of mineral composition, microstructure, and mechanical parameters of the specimens with temperature. Although scholars have studied the patterns of infrared radiation temperature increase during the destabilization and failure of coal-rock, many existing papers focus solely on infrared radiation under laboratory conditions. There is relatively little research on the application of infrared radiation in the field, and relevant literature is also scarce.

A significant amount of research has been conducted by previous scholars on roof blasting, but further refined studies are needed on the prevention and control technologies and parameter settings for different types of hard and thick roof strata. Coal mines currently rely more on micro-seismic monitoring technology to analyze the characteristics before and after roof blasting, with less research on the changes in thermal infrared radiation temperature before and after roof blasting in coal mines. This paper takes the 418 working face at the Chenjiashan coal mine in Shaanxi, China, as its actual research background. It designs the roof blasting parameters for the hard roof of this working face and analyzes the effects using a borehole viewer, an infrared Thermal camera, and an optical fiber camera. The research findings offer insights into the prevention and control of coal bursts in coal mines.

## Overview of the working face

**Fig. 1:**
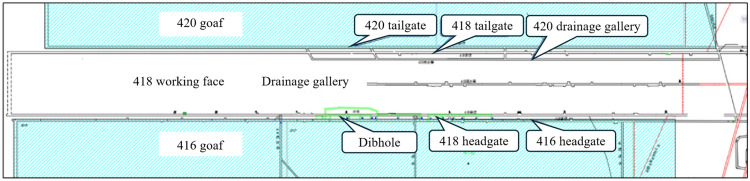
418 Working face layout.

The 418 working face is located in the west wing of the Fourth Mining Area, with the main system of the Fourth Mining Area to the northeast, the goaf of the 416 working face to the southeast, the thin coal area and the mine boundary to the southwest, and the goaf of the 420 working face to the northwest. The longwall retreat mining method with top coal caving is adopted, and the goaf adopts the full collapse method to manage the roof. The designed strike length of the 418 working face is 1875 m. Due to the thinning of the coal seam, the actual strike length of the working face is 1695 m, with a dip length of 154 m. The 418 working face is mining the 4–2 coal seam, which has a significant undulation in the bottom surface. The dip and pitch angles of the coal seam reach up to 7° and 9°, respectively. The elevation of the bottom plate is between 910 and 1037 m, with a burial depth ranging from 265 to 510 m, as shown in Fig. 1.

## Design of roof breaking blasting scheme

### Blasting pre-cracking position

**Fig. 2:**
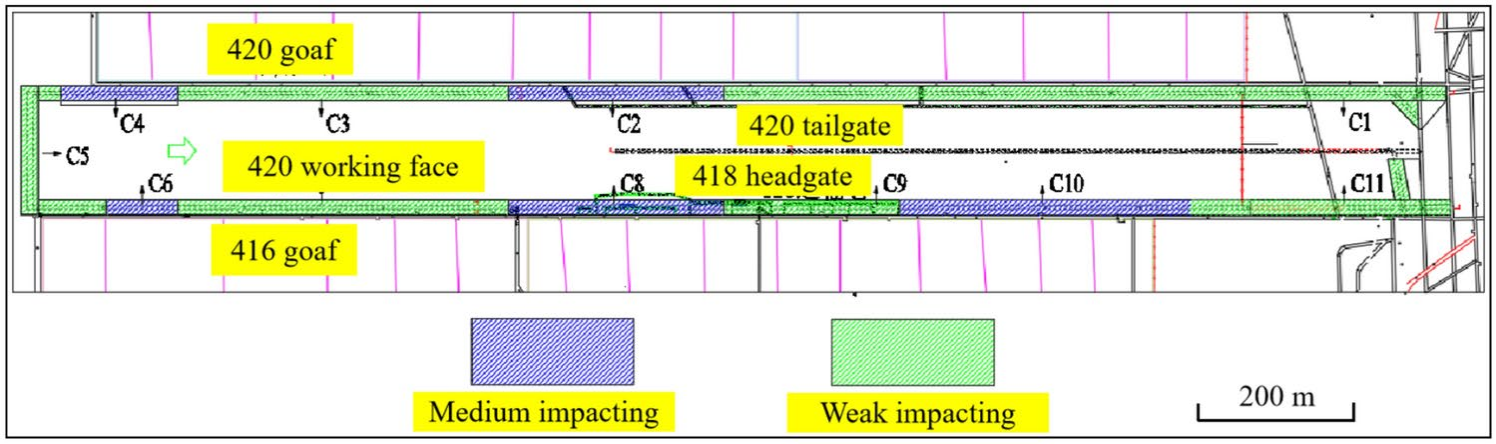
Workface retreat impact hazard zone division diagram.

According to the report ‘Prevention and Control Design for coal bursts in the 418 Fully Mechanized Caving Working Face of Chenjiashan Coal Mine’, the 418 fully mechanized caving working face is divided into 11 coal burst hazard areas, including 6 areas with low coal burst hazard and 5 areas with moderate coal burst hazard. The division of hazard areas is shown in Fig. 2. Based on the different divisions of hazard areas, this paper designs different blasting hole parameters.

### Design of pre-cracking drilling parameters for roof breaking blasting

The blasting design parameters for different coal burst hazard areas are shown in Table 1.

**Table 1:** Comparison of blasting parameters for different areas.

Area	Design scheme	Borehole angle	Hole depth (m)	Charge length (m)
First square area	Scheme I	60°, 45°	36, 45	18, 24
Medium risk area	Scheme II	60°, 45°	60, 70	38, 40
Weak risk area	Scheme II	60°, 45°	60, 70	38, 40
Initial discharge area	Scheme III	85°	12	6
Working face strike area	Scheme IV	20°, 5°	16	8

(1) First square area

During the initial mining period of the 418 working face, when working face reaches the 420 interconnection position and continues to advance close to the length of the working face, first square" phenomenon occurs and the overlying rock strata moves synergistically, which is prone to cause strong mining pressure manifestations. This is because the fracture height of the rock roof layer reaches its maximum, the activity of the roof intensifies, and the degree of danger from strong mining pressure also increases. At this time, a pre-cracking design is proposed for the coarse sandstone (with a vertical height of about 30 m) at 7.65 m in the 893 borehole, and pre-cracking design scheme one is presented.

**Fig. 3:**
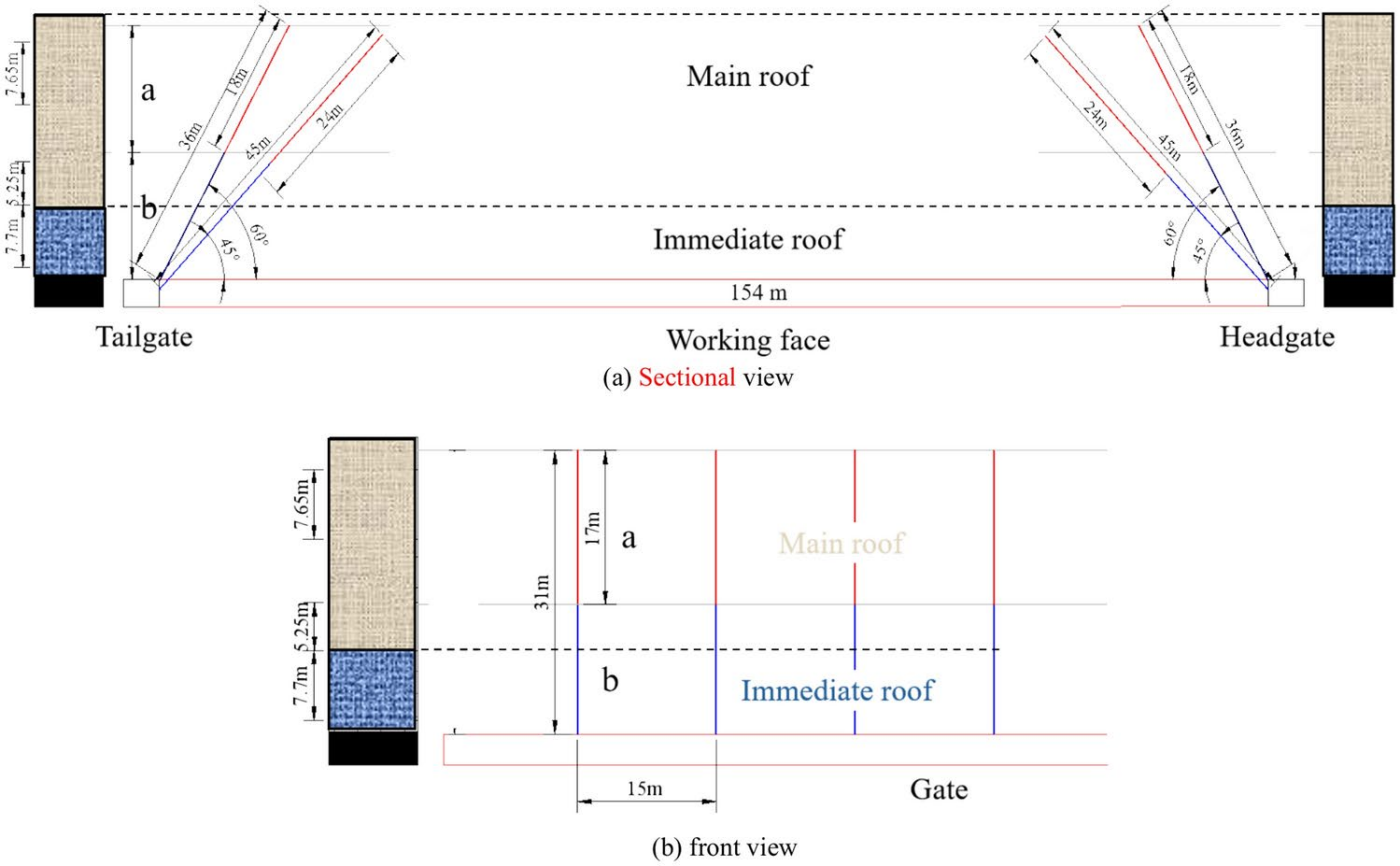
Blasting hole parameters in the first square area of the face.

Considering actual mining operations, the drilling is initiated 100 m ahead of the interconnection, with pre-splitting blasting holes for top coal caving designed at intervals of 15 m. The blasting holes are arranged on the inner side of the working face, with four top coal caving pre-splitting blasting holes per set (two in the head gate and two in the tail gate), each inclined at angles of 60° and 45° to the horizontal plane. The hole diameter is 89 mm, with corresponding hole depths of 36 and 45 m. The lengths of the explosive charges are 18 m and 24 m, respectively, and the sealing length is no less than one-third of the hole depth, as illustrated in Fig. 3. In Fig. 3, 'a' represents the length of the charge, and 'b' represents the sealing length, distinguished by red and blue lines, respectively.

(2) Medium risk area

When the 418 working face is mined to areas with moderate impact risk, it is greatly affected by the burial depth and hard thick roof, and the roof of the working face is prone to accumulate high elastic energy. Based on the investigation of the blasting and pressure relief status of coal mining faces in the surrounding mining areas and the comprehensive analysis of previous drilling data, the vertical height of the main control rock layer is determined to be about 50 m for pre-cracking design, and the pre-cracking design scheme II is proposed.

**Fig. 4:**
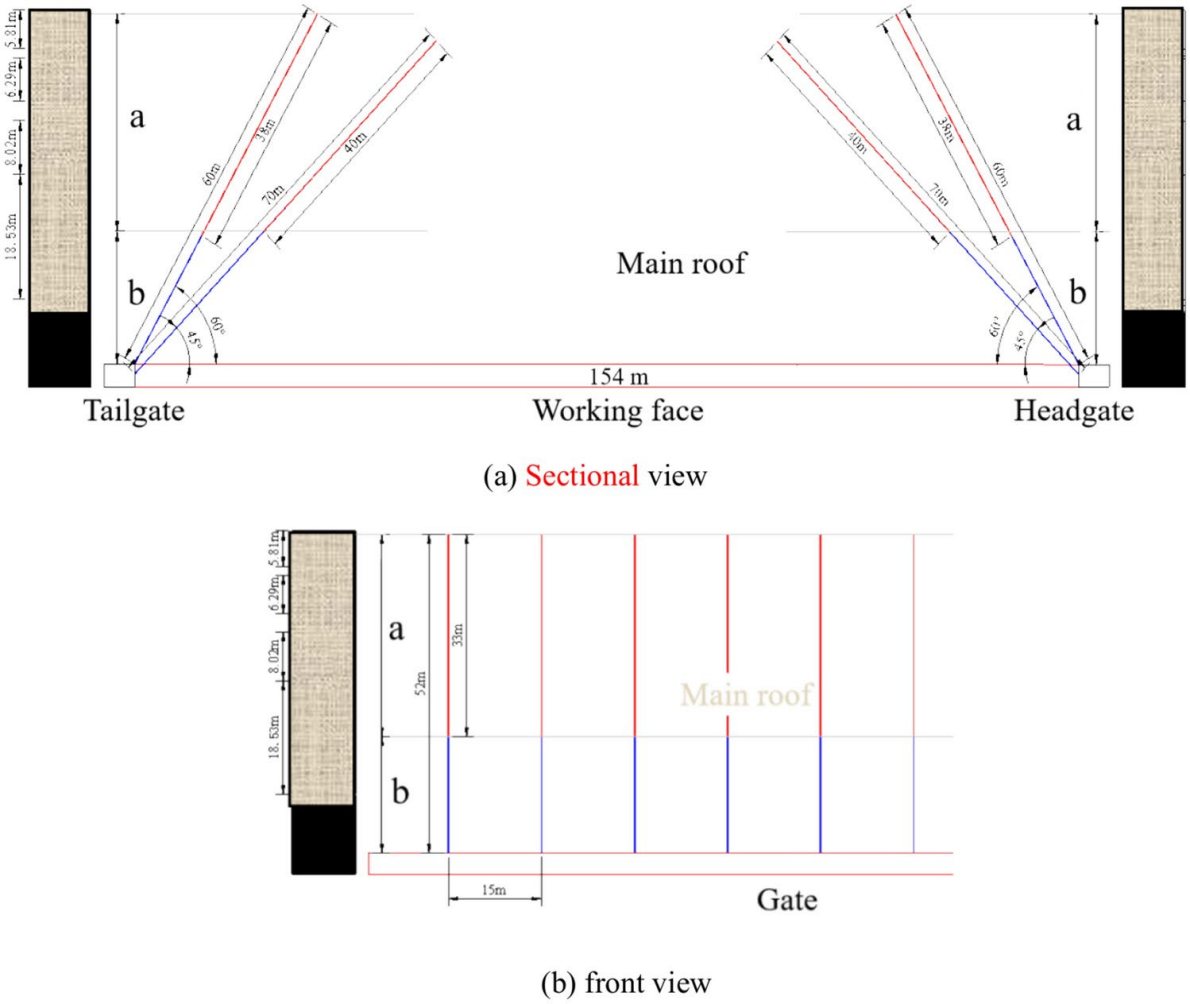
The medium impact danger area of the working face tends to the roof blasting hole parameters.

Design inclined roof pre-cracking blasting holes with a spacing of 15 m. The blasting holes are arranged towards the inner side of the working face, with 4 roof pre cracking holes (2 for the head gate and 2 for the tail gate) arranged in each group, at angles of 60° and 45° to the horizontal plane, with a hole diameter of 89 mm, corresponding to hole depths of 60 m and 70 m, and charge lengths of 38 m and 40 m, respectively. The sealing length of the hole is not less than one-third of the hole depth, as shown in Fig. 4. In Fig. 4, 'a' represents the length of the charge, and 'b' represents the sealing length, distinguished by red and blue lines, respectively.

(3) Weak risk area

When the working face is mined to areas with weak impacting area, the strike drilling still adopts the blasting pre-crackting design scheme II for construction, but a set of strike blasting pre-cracking drilling holes is constructed every 20 m.

(4) Initial discharge area

**Fig. 5:**
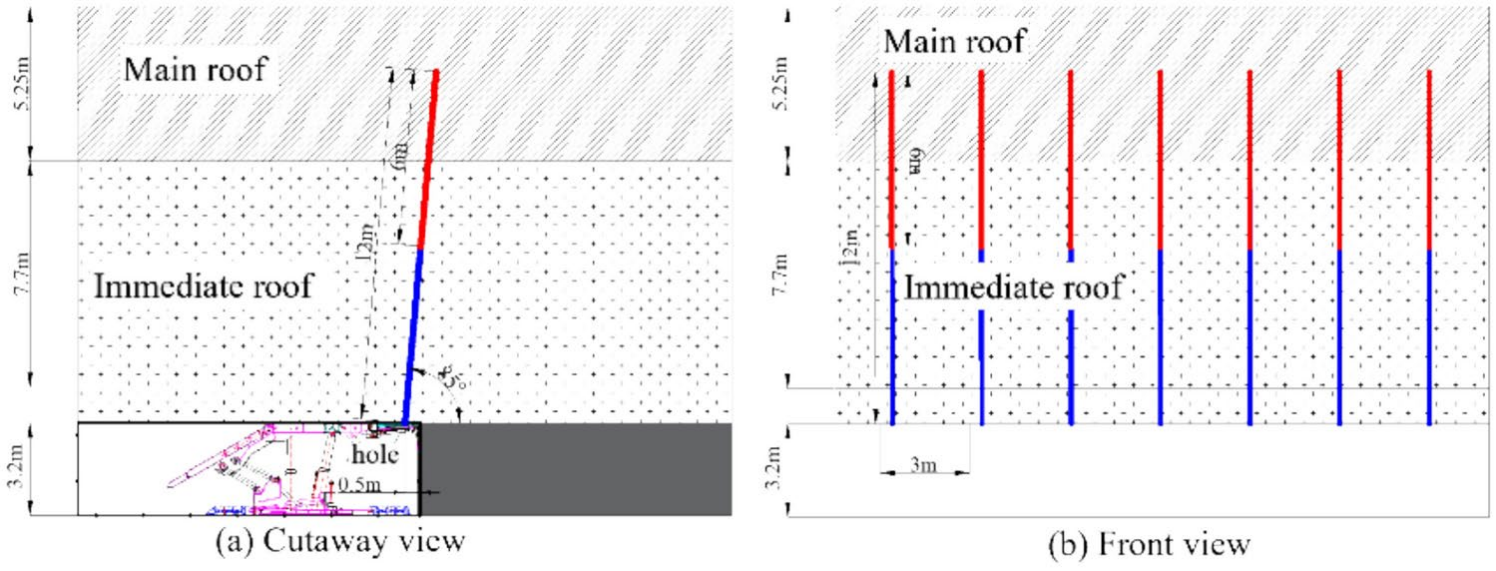
The roof blasting hole parameters in the initial discharge area of the working face.

To ensure the safe and smooth progress of the initial mining and releasing operations in the 418 fully-mechanized caving face, to prevent the large-area hanging roof in the goaf and to reduce the severity of the initial pressure, supplementary design of roof fracturing blasting holes is proposed in the initial mining area of the 418 working face. The blasting horizon is the 7.7 m thick coarse sandstone and the 5.25 m thick siltstone roof in the 893 borehole, and pre-cracking design scheme III is proposed. The blasting holes are arranged on the coal wall side of the working face, with a shot spacing of 3 m. The holes are 0.5 m away from the coal wall, at an angle of 85° to the horizontal plane, with a hole diameter of 89 mm, a hole depth of 12 m, a charging length of 6 m, and a sealing length not less than one-third of the hole depth, as shown in Fig. 5.

During blasting, measures such as installing individual supports under the front beam of the support are taken to protect the support and the front scraper conveyor. If, within a normal advanced distance of 20 m in the working face, the 7.7 m thick coarse sandstone and the 5.25 m thick siltstone roof have already caved, this scheme will not be implemented.

Furthermore, if the working face reaches the normal initial pressure step distance but the initial pressure collapse has not yet occurred, the transport gallery and return air entry of the working face will use Scheme I for strike fracturing, with a set of blasting pre-splitting holes constructed every 10 m.

(5) Working face strike area

In areas with weak impacting area where the coal thickness is less than 6 m (including the first square areas), use Scheme IV (drilling an inclined hole of 16 m with an explosive length of 8 m), and arrange one strike hole every 10 m. The holes are inclined at an angle of 20° towards the direction of advancement along the strike direction and are inclined at an angle of 5° towards both sides of the goaf (on the side of the coal pillar coal wall) in the dip direction. The inclination angle towards the goaf on the side of the coal pillar can be adjusted according to the drilling inspection. The explosive section is half the depth of the drilling hole, and the sealing section is also half the depth of the drilling hole, as shown in Fig. 5.

## On site data effect verification

### Borehole observation instrument data analysis

The borehole observation instrument has the advantages of being intuitive and fast and is widely used in the development of rock mass fractures, weak planes, and other structural conditions, as well as in the evaluation of the integrity of the surrounding rock^28,29^. The device processes the data observed after blasting to determine the development and expansion patterns of roof fractures and delamination. Based on the degree of roof fracture development observed after the borehole inspection, the roof is divided into three zones: the roof intact area, the roof fracture development area, and the roof fragmentation area. Given the similarity in borehole observation results across different risk areas and considering that the medium risk area is the most dangerous area in the 418 working face, this paper analyzes borehole data from the medium risk area of the head gate.

(1) Roof intact area

**Fig. 6:**
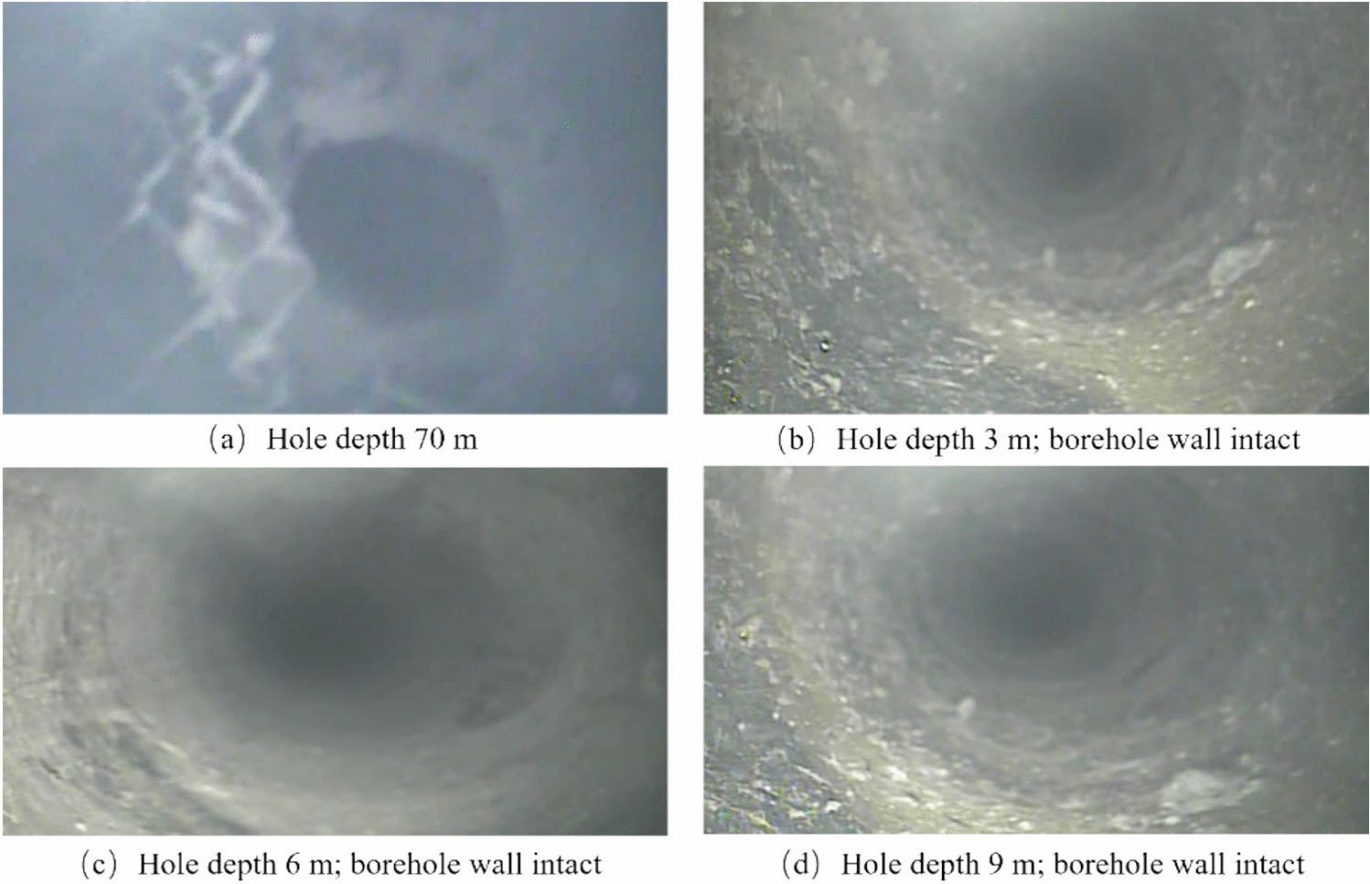
Roof borehole observation for intact areas.

The drilling depth in this area ranges from 0 to 10 m, corresponding to a vertical depth of 0 ~ 5.7 m, of which the hole depth from 0 to 2.1 m is siltstone, and from 2.1 to 10 m is coarse sandstone. The characteristic of this area is that the hole wall is smooth and intact, with no obvious fractures. The development of roof fractures and the expansion rules of delamination are shown in Fig. 6.

According to the imaging results of the roof drilling, when the inspection depth is 3 m, the hole wall is smooth and intact without fractures; at a depth of 6 m, the rock type changes from siltstone to coarse sandstone, and the hole wall is intact without fractures; at a depth of 9 m, the rock type is coarse sandstone, and the hole wall is intact without fractures.

(2) Roof fracture development area

**Fig. 7:**
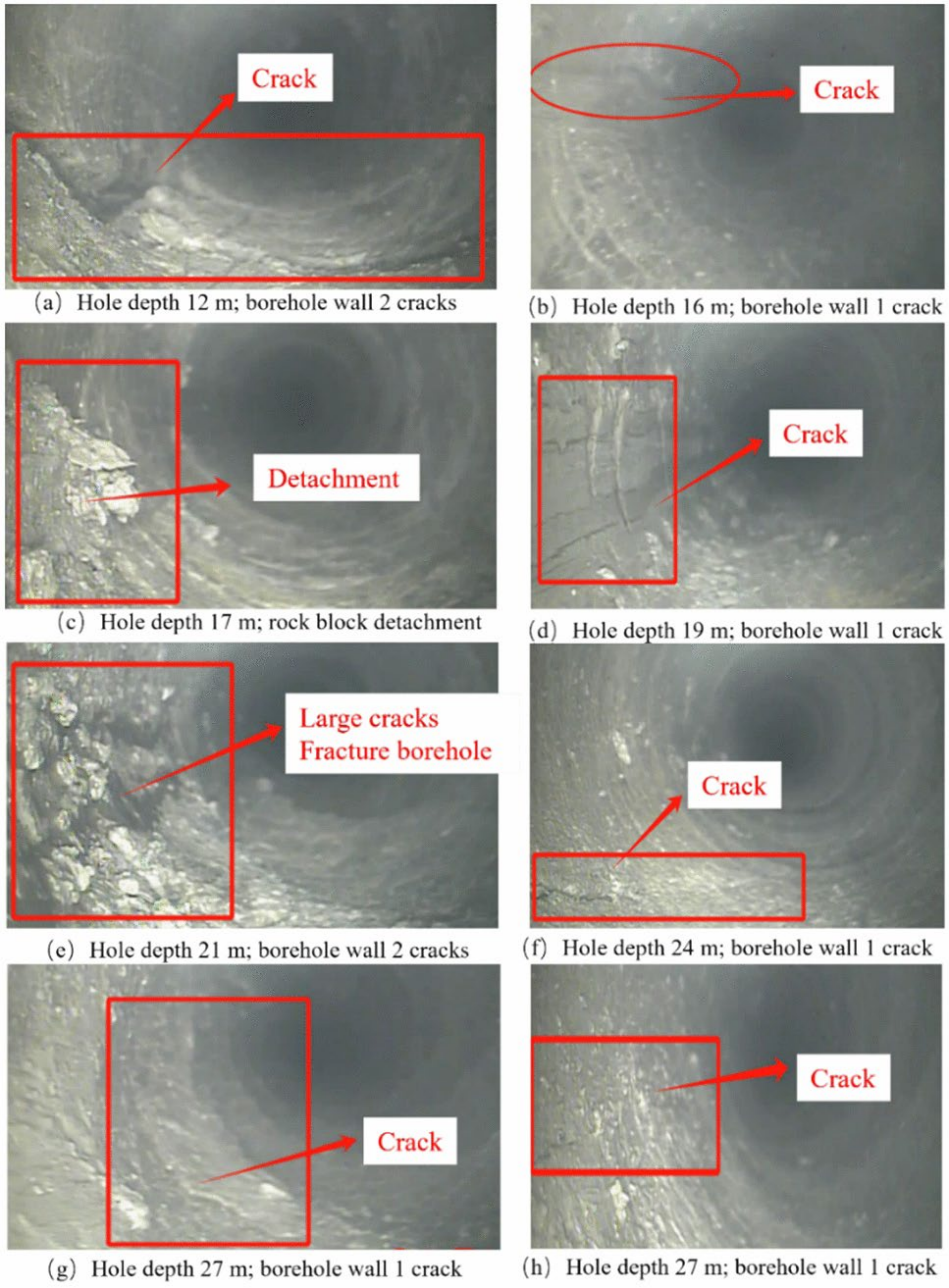
Roof borehole observation for fracture development areas.

The drilling depth in this area ranges from 10 to 33 m, corresponding to a vertical depth of 5.7 ~ 18.9 m. Within this range, the hole depth from 10 to 15.6 m is coarse sandstone, from 15.6 to 24.8 m is siltstone, from 24.8 to 27.7 m is gravelly coarse-grained sandstone, and from 27.7 to 33 m is siltstone. The section from 17 to 33 m is considered the (sub)critical layer. The characteristic of this area is the initial appearance of fractures influenced by factors such as the rock properties themselves and the blasting of the overlying rock. The development of roof fractures and the expansion rules of delamination are shown in Fig. 7.

According to the imaging results of the roof drilling, at a depth of 12 m, the rock type is coarse sandstone, and there are two fractures on the hole wall. One fracture has an opening of about 8 mm and forms a 30° angle with the horizontal direction. The other fracture has a smaller opening and is perpendicular to the direction opposite to the drilling. At a depth of 16 m, the rock type is siltstone with argillaceous cementation, and there is one fracture parallel to the drilling direction on the hole wall with an opening of about 2 mm. At a depth of 17 m, the rock type is siltstone with rock fragments from the hole wall.

At a depth of 19 m, the rock type is siltstone with argillaceous cementation, and there are four fractures parallel to the drilling direction on the hole wall. At a depth of 21 m, the rock type is siltstone, and there are two fractures parallel to the drilling direction on the hole wall with a larger opening. The surrounding rock is severely crushed, which is related to the poor sorting of this rock type. At a depth of 24 m, the rock type is siltstone, and there is one fracture close to the horizontal direction on the hole wall with an opening of about 1mm. At a depth of 27 m, the rock type is gravelly coarse-grained sandstone, the hole wall is relatively rough, and there is one fracture perpendicular to the drilling direction with an opening of about 1 mm. At a depth of 30 m, the rock type is in the transition stage from siltstone to gravelly coarse-grained sandstone, and there is one fracture parallel to the drilling direction with an opening of about 0.5 mm.

(3) Roof fragmentation area

**Fig. 8:**
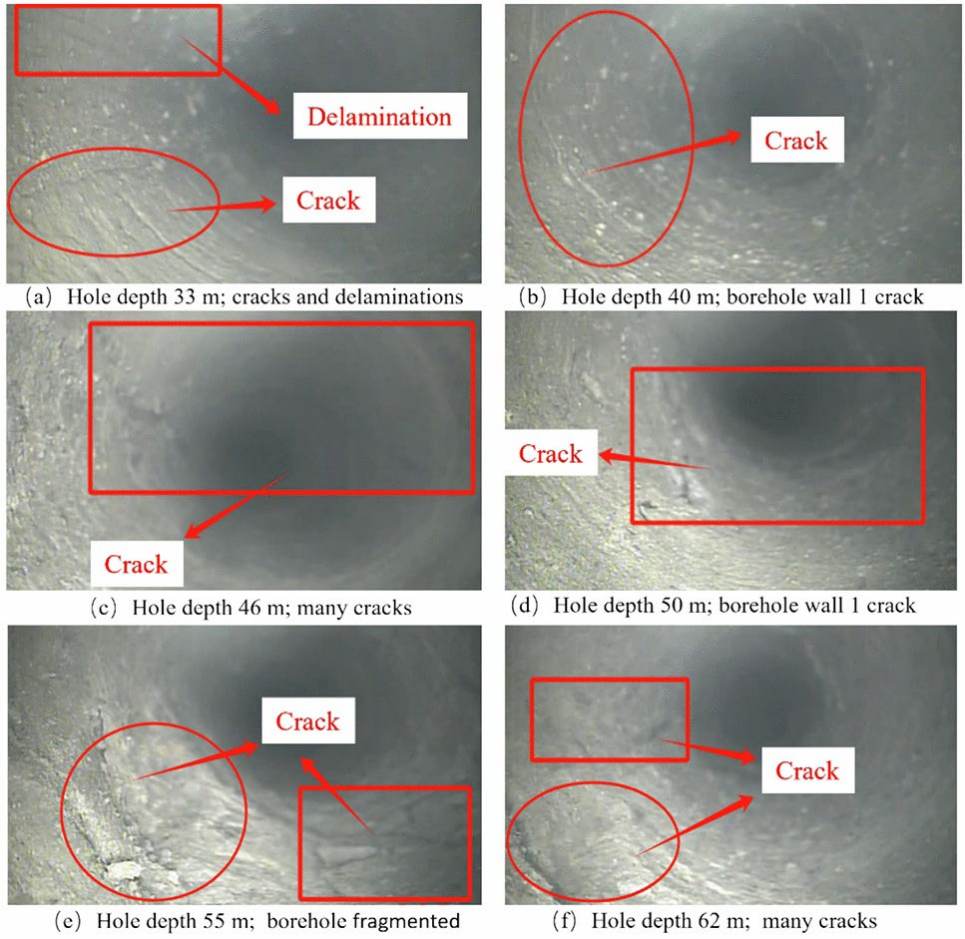
Roof borehole observation for fractured areas.

The drilling depth in this area ranges from 33 to 70 m, corresponding to a vertical depth of 18.9 ~ 40 m. Within this range, the hole depth from 33 to 37 m is fine sandstone, from 37 to 49.6 m is gravelly coarse-grained sandstone, from 49.6 to 56.4 m is siltstone, and from 56.4 to 70 m is conglomerate. The characteristic of this area is that the drilling fractures are relatively well-developed, mostly consisting of intersecting and penetrating fractures. The intersection areas have broken hole walls and are greatly affected by blasting. The development of roof fractures and the expansion rules of delamination are shown in Fig. 8.

According to the imaging results from the roof drilling, at a depth of 33 m, the rock type is fine sandstone, the hole wall is relatively rough, with delamination, one fracture parallel to the drilling direction, and one fracture perpendicular to the drilling direction; at a depth of 40 m, the rock type is gravelly coarse-grained sandstone, the hole wall is relatively rough, with one fracture perpendicular to the drilling direction and a small opening; at a depth of 46 m, the rock type is gravelly coarse-grained sandstone, with one fracture forming a 55° angle with the horizontal direction, penetrating through the drilling, and a small opening; at a depth of 50 m, the rock type is siltstone, with one fracture forming a 65° angle with the horizontal direction, and an opening of about 2 mm; at a depth of 55 m, the rock type is siltstone, with higher hardness, two well-developed fractures on the hole wall, one intersecting perpendicularly, the rock at the intersection of the fractures is relatively fragmented; at a depth of 62 m, the rock type is conglomerate, with higher hardness, two developed fractures on the hole wall, one parallel to the drilling direction with an opening of about 0.5 mm, and one perpendicular to the drilling direction with an opening of about 4 mm.

In summary, the surrounding rock is relatively intact at depths less than 10 m, after which fractures begin to develop. As the depth continues to increase, the fractures increase and their development extent expands, with delamination occurring at 30 m. The degree of destruction from 40 to 70 m is greater than at 30 m, with an increasing number of longitudinal fractures and more instances of longitudinal fractures penetrating through.

### Infrared monitoring effect verification

#### Principles of infrared radiation monitoring

After rocks are subjected to loads, the internal stress levels will significantly increase, and the original cracks and fissures within the rocks gradually close. Subsequently, as the load is gradually increased, new cracks begin to germinate, expand, and penetrate, and the development of rock cracks will affect its infrared radiation response information^30,31^. During the construction process of mining faces, if coal and rock experience fractures and other defects, there will be abnormal changes in the infrared radiation of the rock surface.

The core of infrared thermography technology lies in using an infrared thermal camera to convert invisible infrared radiation into visible digital images. The infrared thermal camera collects the electromagnetic waves of the infrared radiation emitted by objects through an optical infrared lens, determines the intensity of the infrared radiation through its internal components, and finally uses the specialized processing software of the infrared thermal camera to convert the intensity of the infrared radiation into infrared radiation temperature. This results in the infrared radiation temperature of each pixel point, and combining this with infrared recording is the process of infrared thermography^32^.

Unlike traditional microseismic monitoring, which focuses on discrete acoustic signals generated by rock fractures, infrared thermography dynamically tracks the thermal effects of energy release during rock fracturing by capturing surface temperature changes in coal and rock masses. When internal cracks initiate and propagate in rocks, their surface infrared radiation temperature exhibits abnormal responses due to changes in stress state. Such thermal anomalies often appear earlier than detectable microseismic signals and can continuously and non-contactly visualize the temperature field distribution of large-scale coal-rock masses.

(1) Infrared monitoring equipment

**Fig. 9:**
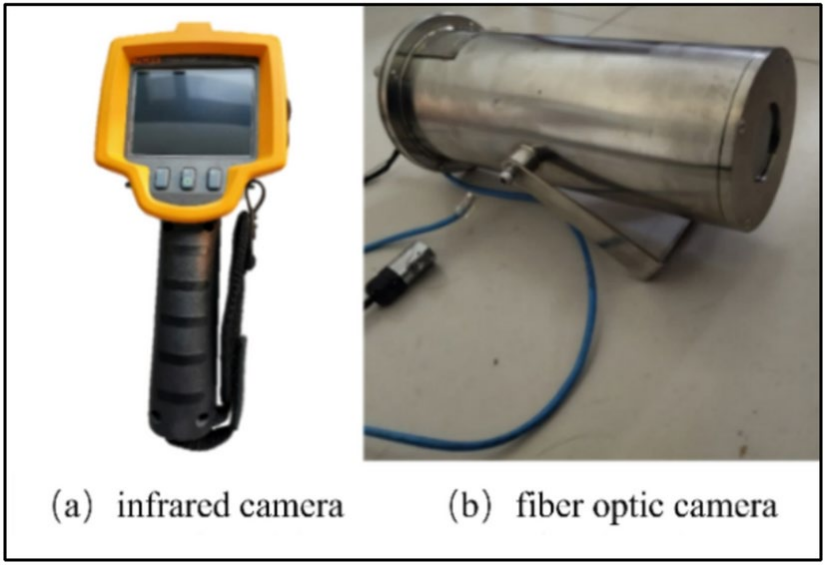
Infrared thermal imaging temperature measurement equipment.

To enhance the accuracy of infrared detection, this paper utilizes the Ei60 handheld infrared thermal camera (Fig. 9a) and the KBA153(A) mine intrinsically safe optical fiber camera(Fig. 9b) to conduct non-destructive monitoring of the infrared radiation temperature in the 418 working face’s extraction gallery.

(2) Monitoring point layout method

**Fig. 10:**
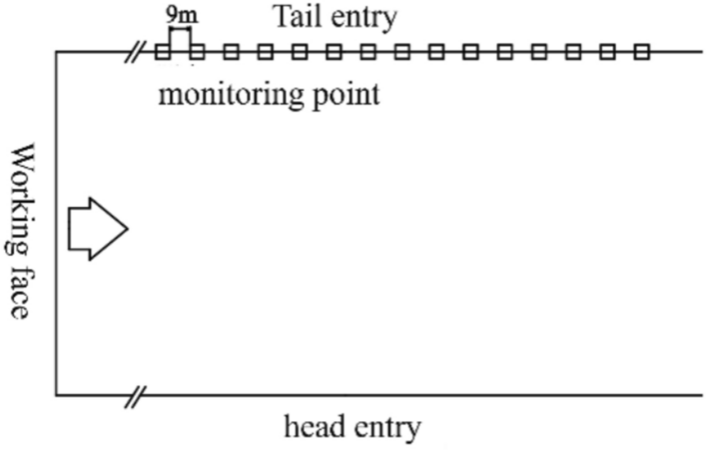
Schematic diagram of measuring point layout.

To achieve non-destructive infrared monitoring of the stability of the surrounding rock structure in the roadway, considering the actual production work on site, 15 monitoring points were determined in the return airway of the 418-working face before monitoring, with a spacing of 9 m between each point. At each monitoring station, a 1 m^2^ area was marked with spray paint to serve as an effective monitoring area for data extraction and analysis. During the observation period, the thermal camera was approximately 1 m away vertically from the observation area. Inside the monitoring points, there were no abnormal heat sources such as mechanical equipment, except for coal and metal mesh, to ensure the accuracy of the detection results. The layout of the monitoring points is shown in Fig. 10.

#### Infrared radiation monitoring results

(1) Infrared thermography analysis

By acquiring thermal images of the coal and rock surfaces at the site using an infrared thermal camera, it is possible to reflect the migration characteristics of thermal infrared radiation anomalies in the development process of fractures in the surrounding rock of the roadway under stress disturbance. When coal and rock fracture, thermal infrared radiation anomalies will appear in the corresponding fracture or location. To ensure the accuracy of the measured data, an adaptive median filtering method is used to filter the infrared thermal images, and the computer is utilized for reconstruction.

**Fig. 11:**
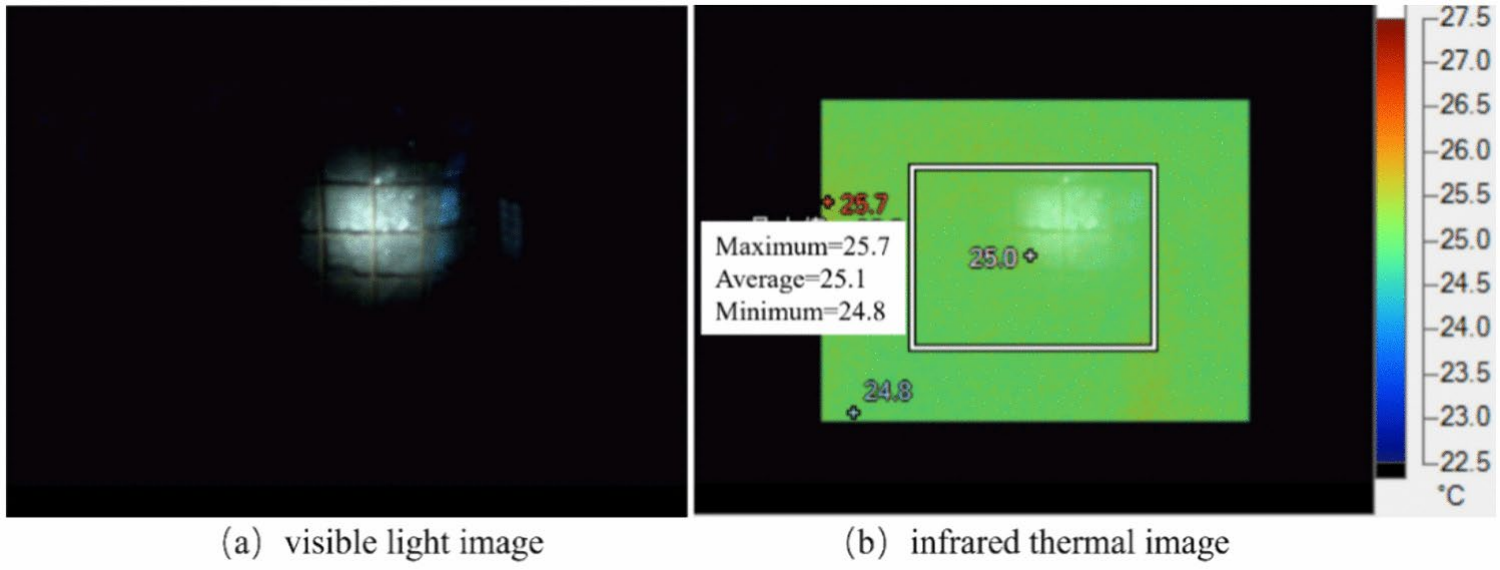
Visible light image and infrared thermal image of monitoring point 4# in the working face tail gate.

Figure 11 shows the visible light image and infrared thermal image of the 4# monitoring point in the tail gate of the 418-working face on October 1, 2023. At this time, the 4# monitoring point is 360 m away from the 418 working face and 94 m away from the roof-cutting blasting position. The corresponding infrared thermal image does not show any significant thermal infrared radiation anomalies. This indicates that there are no large-scale macroscopic fractures within the coal body, and the impact of mining activities and the effects of the roof-cutting blasting have not reached the 4# monitoring point, suggesting that the coal seam in this area is stable.

**Fig. 12:**
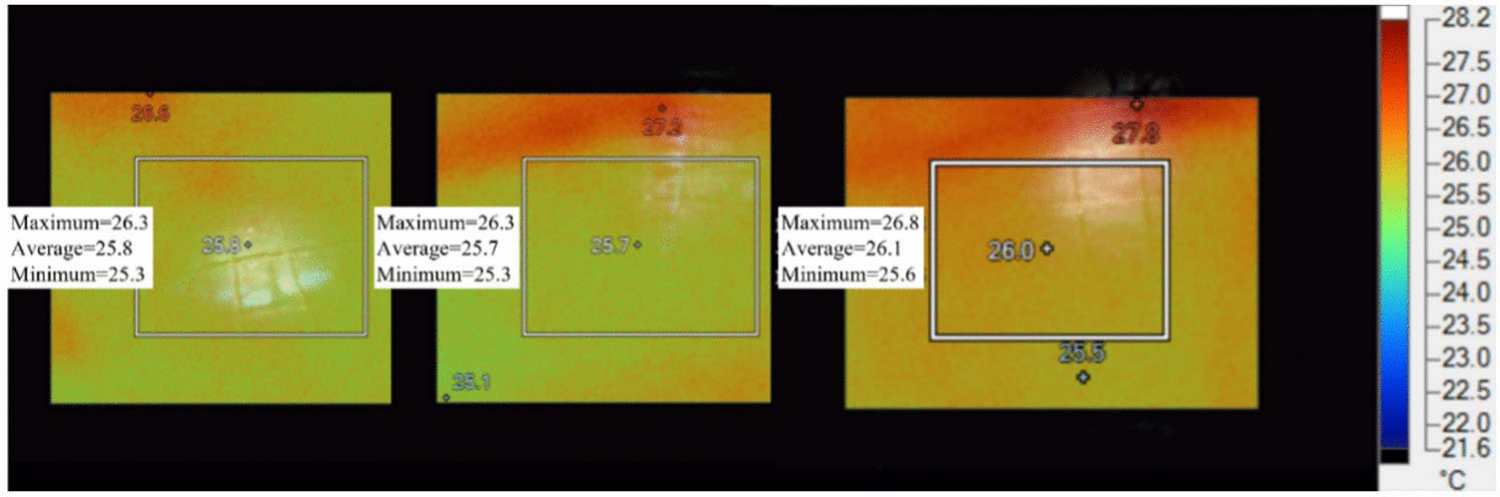
Infrared thermal image of 4# measuring point of tail gate on 418 working face.

Figure 12 shows the infrared thermal images of the 4# monitoring point taken at different times within one day after the blasting on November 16, 2023. At this time, the 4# monitoring point is 178 m away from the 418 working face and 44 m away from the roof-cutting blasting position. It can be observed that a high-temperature area appears at the 4#, and both the range and temperature of the abnormal area have increased. The maximum value of the high-temperature area has risen by 1.2°C, and both the average temperature and the minimum temperature have increased by 0.3°C. Excluding the temperature changes at the monitoring point caused by environmental temperature variations, the stress from the overlying rock layers after the roof-cutting blasting is transmitted to the coal body, causing the coal to be compressed. The closure of existing joints and the formation of new joints reflect changes in the infrared thermal imaging information.

(2) Temporal-spatial evolution features of infrared radiation

The Average Infrared Radiation Temperature (AIRT) can very intuitively reflect the overall strength of the infrared radiation field on the surface of coal and rock, reflecting the important characteristics of infrared radiation changes. The AIRT can be used as a quantitative analysis indicator to reflect the characteristics of infrared radiation changes in loaded coal and rock. The calculation formula is as follows:

1 $$AIRT\left(P\right)=\frac{1}{{L}_{x}}\frac{1}{{L}_{x}}\sum_{x=1}^{{L}_{x}}\sum_{y=1}^{{L}_{y}}{f}_{p}(x,y)$$

where as: AIRT(*p*) represents the average infrared radiation temperature of the *p*th frame in the thermal image sequence; *f*_*p*_(*x*,*y*) is the infrared radiation temperature matrix of the *p*th frame in the infrared thermal image sequence; *L*_*x*_ is the maximum number of rows and columns for *x*; *L*_*y*_ is the maximum number of rows and columns for *y*.

During the long-term monitoring process, the monitoring point is affected by mining activities and roof-cutting blasting, leading to local fractures within the monitoring point. The AIRT can reflect the overall changes on the coal surface but obscures the development of joints and fractures within the monitoring point. Therefore, using the maximum infrared radiation temperature as a stress indicator for the coal mass can illustrate the development of joints and fractures within the monitoring point.

**Fig. 13:**
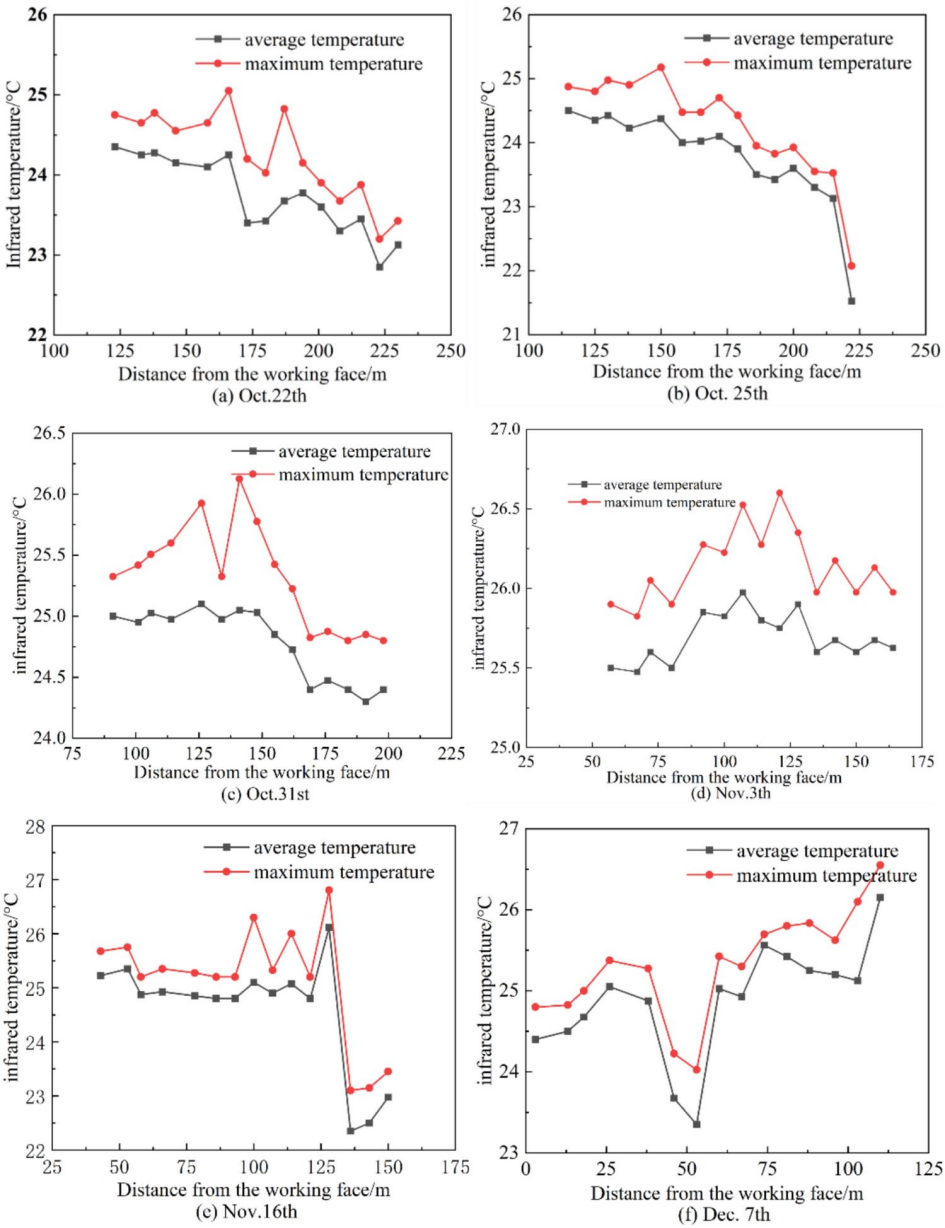
The infrared radiation temperature of each monitoring point varies with the distance of the working face.

Figure 13 shows the curve of infrared radiation temperature on the coal surface at the monitoring points within 8–200 m range from the 418 working face, varying with the distance to the working face. From Fig. 13, it can be seen that in the 0–50 m range from the 418 working face, due to the stress increase area affected by mining activities, the coal mass is under the maximum stress, and the infrared radiation temperature reaches a peak value, which then decreases as the stress decreases. In the 50–150 m range, both the highest and average infrared radiation temperatures show a significant increase, with the maximum increase reaching up to 3 °C, and another peak value is reached at the 150 m range, after which the infrared radiation temperature drops to a lower level.

**Fig. 14:**
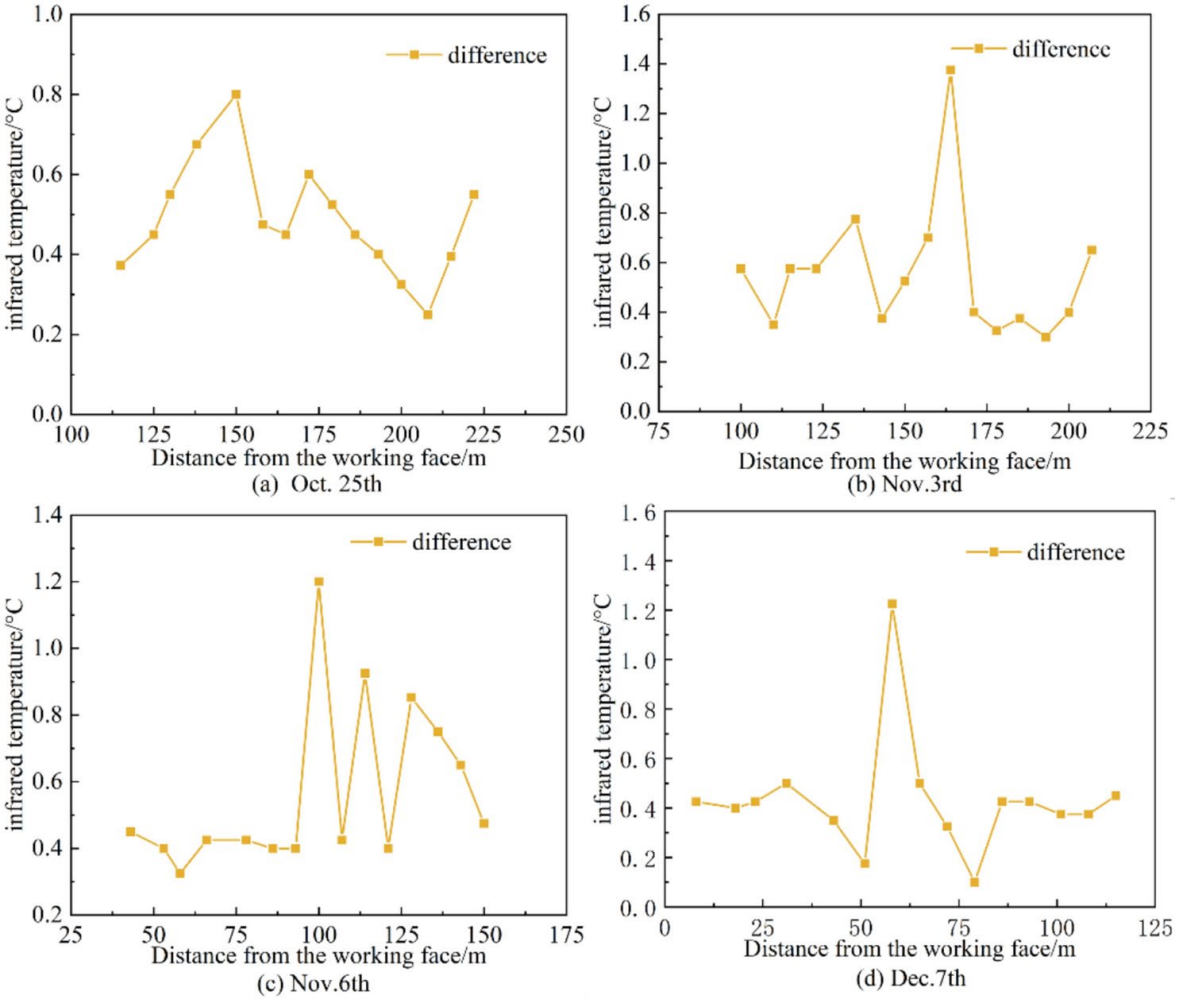
Curve of the difference between the highest and average infrared radiation temperatures at each monitoring point as a function of the distance from the working face.

Figure 14 illustrates the variation curve of the difference between the maximum and average infrared radiation temperatures at various monitoring points in the 418-working face over different periods, with respect to the distance from the working face. As can be seen from Fig. 14, the difference between the maximum infrared radiation temperature and the average infrared radiation temperature peaks near 30 m from the working face and then decreases, reaching another peak near 150 m from the working face, with other monitoring points showing a difference of 0.2–1.2 °C. The two peaks indicate that there are significant joint fractures in the coal mass within these two ranges due to the development underload. The locations of the two peaks coincide with the stress increase area affected by mining activities and the roof-cutting blasting area.

Within the 0–200 m range in front of the working face, looking at the spatial distribution pattern of infrared radiation temperature and stress online monitoring results, the two parameters show the same trend in space. The change in the average infrared radiation temperature of the coal mass can reflect the change in stress above the coal mass. The peak in infrared radiation temperature at the roof-cutting blasting location indicates that after the implementation of the roof-cutting blasting, the stress in the corresponding area of the key layer is released and transmitted to the coal mass.

### Fiber optic camera monitoring results

On October 22, 2023, a roof cutting blasting operation was carried out in the tail gate. Before the construction, observation points were set up near the blasting site. The KBA153(A) mining optical fiber camera was used to monitor the infrared radiation information of the coal body after blasting. Throughout the monitoring process, all personnel were evacuated from the return airway, and all equipment in the airway was shut down, ensuring the accuracy of the monitoring results.

#### Infrared thermal image of measuring points in the tail gate

**Fig. 15:**
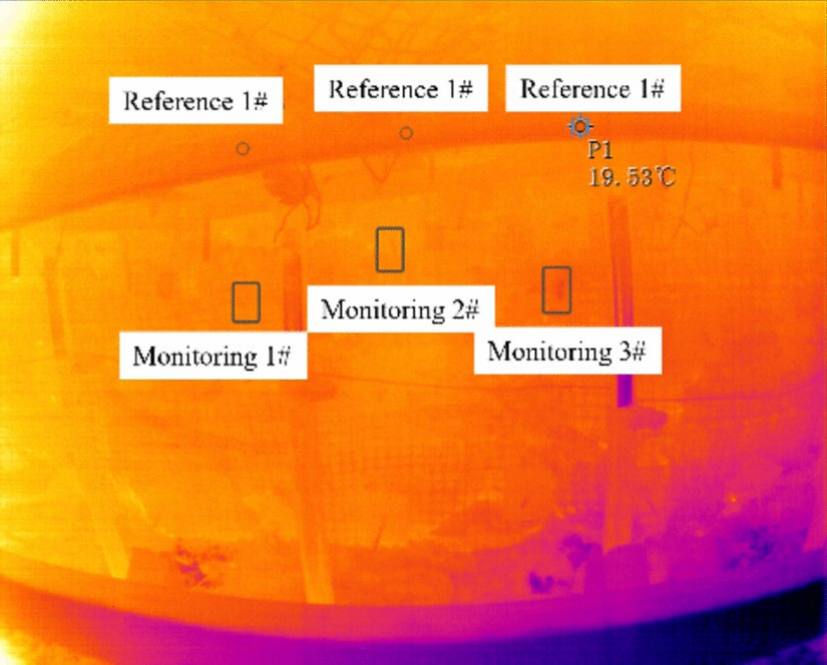
KBA153 (A) intrinsically safe fiber optic camera for mining, infrared thermal image.

As shown in Fig. 15, it is the infrared thermograph of the KBA153(A) mine optical fiber camera. The image includes a single hydraulic prop, a single-rail crane track, metal mesh, and coal body. Throughout the monitoring process, the single-rail crane track is suspended in the tunnel without being affected by external forces, and its temperature change can reflect the change in environmental temperature. Therefore, three reference points are selected on the single-rail crane track, and their average value is taken as the reference value for the temperature change at the monitoring point. Three monitoring points are selected on the coal. The difference between the temperature at the monitoring point and the reference point temperature reflects the temperature change process at the measurement point after the roof cutting blasting, which in turn reflects the stress change process within the coal body.

#### The variation law of infrared radiation temperature after blasting

**Fig. 16:**
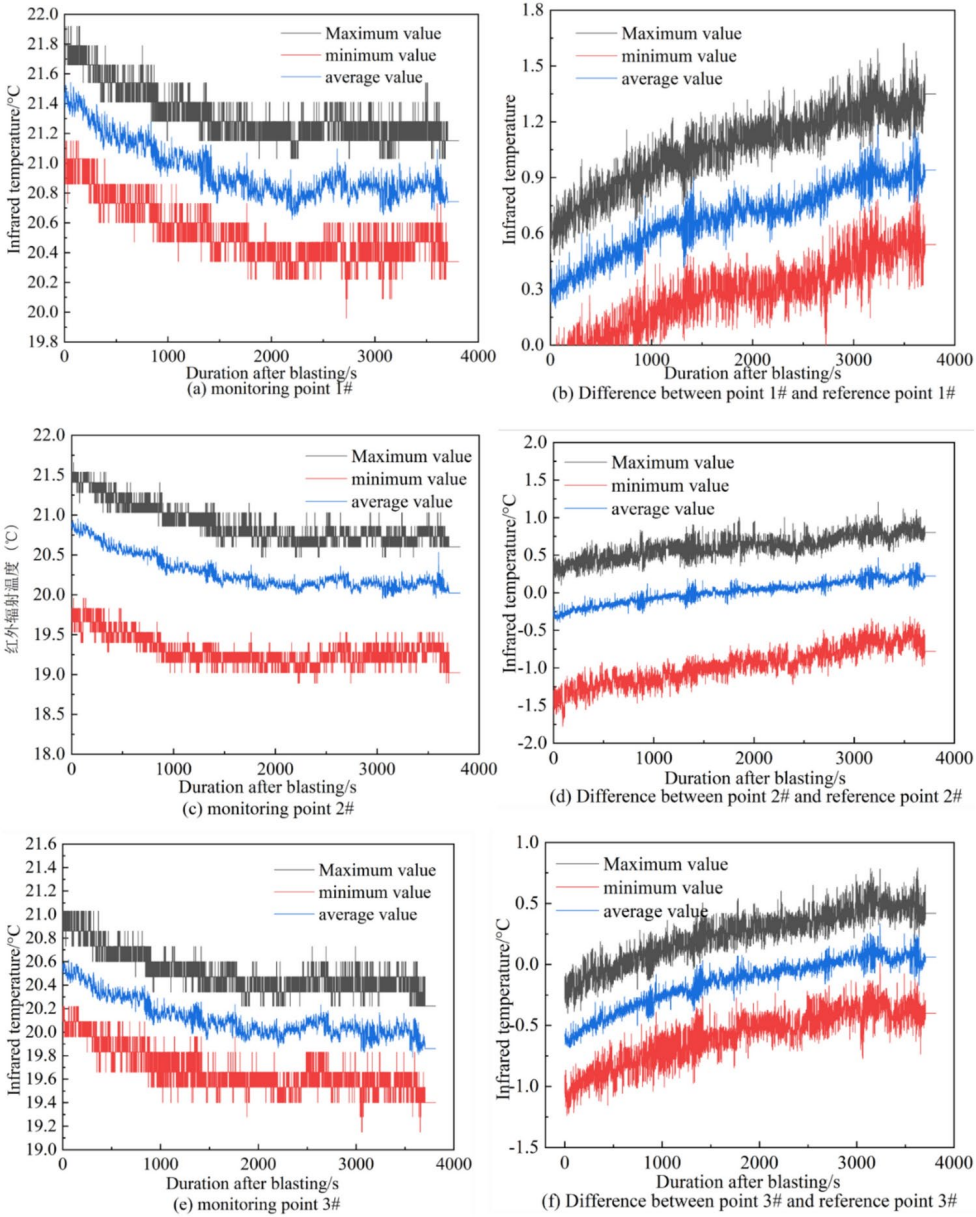
The infrared radiation temperature of each measuring point changes with time.

As shown in Fig. 16, the curve of the average of the highest, lowest, and average infrared radiation temperature values of the three reference points changes with monitoring time. From the image, it can be seen that the temperature of the single-rail crane track decreases over time, indicating that the ambient temperature is declining during that period.

Figure 16 shows the curves of infrared radiation temperature changes over time for various monitoring points. In particular, figures (a), (c), and (e) represent the raw infrared radiation temperatures for Monitoring point 1#, Monitoring point 2#, and Monitoring point 3#, respectively. It can be observed that the infrared radiation temperatures at these three monitoring points generally exhibit a decreasing trend along with the ambient temperature. After subtracting the reference point temperatures, figures (b), (d), and (f) are obtained, which indicate that within one hour after the roof cutting blasting, the temperature at the monitoring points rises to a peak and then decreases, with the overall temperature increase ranging from 0.5 to 0.7°C. The patterns of infrared radiation temperature changes at the three monitoring points match the patterns observed in the laboratory for coal and rock samples under pressure-induced instability, suggesting that after the roof cutting blasting, the stress in the key layers above the coal body is released and transmitted to the coal body, causing changes in the load on the coal body. The infrared radiation temperature shows an increasing trend over time as a result. Once the stress is released, the overlying rock strata on the working face return to a state of equilibrium, and the load no longer changes, causing the monitoring point temperatures to revert to their original values.

## Conclusions

(1) Based on the geological conditions of the 418 working face at the Chenjiashan coal mine, the working face is divided into weak impact coal burst hazard areas and moderate impact coal burst hazard areas. Blasting construction plans have been designed for the First square area, medium risk area, weak risk area, initial discharge area and working face strike area.

(2) Data from on-site borehole inspections indicate that after blasting, a large number of fractures and delaminations occurred, with several fractures further developing into delaminations and localized fractured zones appearing, which proves the formation of a “buffer zone” in the roof and floor strata. This has achieved pre-splitting of thick and hard roofs, reduced the degree of stress concentration, and had a significant pressure relief effect.

(3) Long-term infrared radiation monitoring was conducted on the tail gate of the 418 working face after the roof cutting blasting. The results showed that near the blasting point, the infrared radiation temperature monitoring indicated that within one hour after the implementation of the roof cutting blasting, the coal body at the roof cutting position experienced a process of temperature rise followed by a decrease, with the temperature at the monitoring point increasing by 0.5–0.7 °C. After the roof cutting blasting was carried out, the key strata above the working face were destroyed, and the stress was released and transmitted to the corresponding area of the coal mass, causing an increase in stress on the coal mass and an increase in infrared radiation temperature.

(4) Engineering application recommendations: for different coal burst risk zones in isolated island working faces, differentiated blasting schemes should be formulated based on geological characteristics and stress states: enhance pre-splitting in high-risk areas to ensure safety, and optimize parameters in low-risk areas to reduce costs, achieving a balance between safety and economic efficiency. Given the significant field application effect of infrared monitoring in early detection of temperature anomalies on coal-rock surfaces, it is recommended to further combine it with microseismic monitoring technology. By fusing temperature field and vibration field data, the comprehensiveness of coal burst precursor identification and the accuracy of risk prevention can be improved.

## Data Availability

The datasets used and/or analysed during the current study available from the corresponding author on reasonable request.
